# Hydatid cyst in the stomach wall

**DOI:** 10.1590/0037-8682-0598-2020

**Published:** 2021-03-08

**Authors:** Gökhan Polat, Bahar Yılmaz Çankaya, Recep Sade

**Affiliations:** 1 Ataturk University, Medical Faculty, Department of Radiology, Erzurum, Turkey.

**FIGURE 1:**
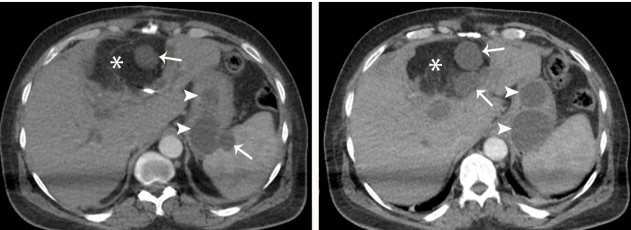
Computed tomography of the abdomen showing multiple cystic lesions in the liver, spleen, and stomach (arrows). Parenchymal loss was observed in the liver due to cystectomy (asterisk). Stomach wall hydatid cysts extend to intracavitary areas (arrowheads).

A 59-year-old man was admitted to our emergency department with abdominal distension and pain complaints. The patient had a history of surgery due to a liver hydatid cyst. (Figure 1, asterisk). Abdominal computed tomography (CT) showed multiple hypodense cystic lesions in the liver operation area and spleen (Figure 1, arrows). Additionally, CT showed two hypodense cystic lesions on the stomach wall. The first cystic lesion measured 2 cm × 2 cm × 2 cm and the second, 3 cm × 3 cm × 3 cm; both extend to the stomach cavity area (Figure 1, arrowheads). Laboratory values showed increased IgE levels at 342 IU/mL. CT findings of the patient were interpreted as liver and gastric hydatid cysts. The diagnosis of hydatid cyst was confirmed by surgery.

The larval form of *Echinococcus granulosus* causes cystic echinococcosis, endemic to several climates in the world^1^. Hydatid cysts usually involve the liver, spleen, and lungs. Stomach hydatidosis is quite rare, with a few cases described in the literature^1^. Hydatid cysts in the gastrointestinal system can be confused with duplication cysts and mesenteric cysts^2^. Hydatid cysts should be considered during the differential diagnosis of gastrointestinal system cysts in endemic areas.
